# Validation of Blood Transfusion Risk Scores (TRACK and TRUST) in a Cardiac Surgery Service in Brazil

**DOI:** 10.21470/1678-9741-2022-0156

**Published:** 2023

**Authors:** Cristiano Berardo Carneiro da Cunha, Verônica Soares Monteiro, Diogo Luiz de Magalhães Ferraz, Rodrigo Mezzalira Tchaick, Jeú Delmondes de Carvalho Júnior, Igor Tiago Correia Silva, Fernando Augusto Marinho dos Santos Figueira, Lívia Barbosa Andrade

**Affiliations:** 1 Department of Cardiovascular Surgery, Instituto de Medicina Integral Professor Fernando Figueira (IMIP), Recife, Pernambuco, Brazil; 2 Department of Cardiology, Instituto de Medicina Integral Professor Fernando Figueira (IMIP), Recife, Pernambuco, Brazil; 3 Department of Cardiovascular Surgery, Hospital Dom Helder Câmara (HDH), Cabo de Santo Agostinho, Pernambuco, Brazil; 4 Department of Post-Graduation, Instituto de Medicina Integral Professor Fernando Figueira (IMIP), Recife, Pernambuco, Brazil

**Keywords:** Area Under Curve, Blood Transfusion, Comprehension, Confidence Intervals, Erythrocytes, Thoracic Surgery, Risk Factors.

## Abstract

**Introduction:**

Transfusion of red blood cells is recurrent in cardiac surgery despite the well-established deleterious effects. Identifying patients with higher chances of requiring blood transfusion is essential to apply strategic preventive measures to reduce such chances, considering the restricted availability of this product. The most used risk scores to predict blood transfusion are the Transfusion Risk and Clinical Knowledge (TRACK) and Transfusion Risk Understanding Scoring Tool (TRUST). However, these scores were not validated for the Brazilian population. The objective of this study was to assess the accuracy of TRACK and TRUST scores in estimating the need for postoperative transfusion of red blood cell concentrates (TRBCC) after cardiac surgery.

**Methods:**

A clinical retrospective study was conducted using the database of a Brazilian reference service composed of patients operated between November 2019 and September 2021. Scores were compared using Mann-Whitney U test. Hosmer-Lemeshow goodness of fit test assessed calibration of the scores. Accuracy was assessed using the area under the receiver operating characteristic curve (AUC). All analyses considered a level of significance of 5%. The study was approved by the research ethics committee (CAAE 55577421.4.0000.5201).

**Results:**

This study assessed 498 patients. Only the TRACK score presented good calibration (P=0.238; TRUST P=0.034). AUC of TRACK was 0.678 (95% confidence interval 0.63 to 0.73; P<0.001), showing a significant accuracy.

**Conclusion:**

Between the scores analyzed, only the TRACK score showed a good calibration, but low accuracy, to predict postoperative TRBCC after cardiac surgery.

## INTRODUCTION

Cardiac surgeries consume considerable amounts of hemoderivatives due to concerns about bleeding and hemodilution during proceedings. The incidence of perioperative blood transfusion ranges between 40% and 90%, depending on duration and complexity of the surgery, pre-existing anemia, and the patient’s age^[^1^,^2^]^. Although blood transfusion is important, knowledge about its deleterious effects is well-established. Studies showed that the need for perioperative blood transfusion during cardiac surgery could increase infection levels and lead to kidney insufficiency, lung complication, or death^[^3^,^4^]^.

Risk scores were created to predict the risk of blood transfusion during cardiac surgery, providing better strategic planning. The two most widespread scores are the Transfusion Risk and Clinical Knowledge (TRACK) and Transfusion Risk Understanding Scoring Tool (TRUST), developed in Italy and Canada, respectively, and published between 2006 and 2009^[^5^,^6^]^.

The difficulty of blood banks in attending to the great demand of hospitals is another important aspect and was aggravated by the coronavirus disease 2019 (or COVID-19) pandemic. For example, safe blood donors reduced by up to 38% in the municipality of Rio de Janeiro compared with the same period of 2019, and this situation may be extrapolated to the entire country^[^7^]^. In this sense, predicting the risk of bleeding improves decision-making, quality control, and allocation of available resources to apply effective prophylactic measures during the perioperative moment (*e.g.*, perioperative red blood cell salvage)^[^1^,^8^-^13^]^.

The Brazilian population presents different characteristics compared with Canadian or Italian populations, such as access to health and nutritional care. Therefore, the validation of these instruments for our population is needed. Thus, this study aimed to assess the accuracy of TRACK and TRUST scores in predicting the need for postoperative transfusion of red blood cell concentrates (TRBCC) after cardiac surgery.

## METHODS

This retrospective clinical study was conducted to validate risk scores for TRBCC. The study was approved by the research ethics committee of the Instituto de Medicina Integral Professor Fernando Figueira (IMIP) (opinion number 5.259.262). The informed consent form was dispensed, considering the use of a secondary database without identifying participants.

Data were collected between October 2021 and December 2021 and included all cardiac surgeries (myocardial revascularization, heart valve surgery, cardiac transplantation, aortic root surgery, and correction of congenital pathologies) conducted between November 2019 and September 2021 at the department of cardiology of IMIP.

The restrictive strategy guided by bedside hemodynamic and gasometric parameters is the standard criterion for blood transfusion in the service. In this strategy, blood transfusion is only suggested when the hematocrit (Ht) value is below 24% from the beginning of the surgery to intensive care unit discharge^[^5^]^.

TRUST and TRACK scores were calculated based on the following variables: age, sex, weight, hemoglobin (Hb), Ht, postoperative creatinine, surgery type (*e.g.*, valvular, myocardial revascularization, aortic root surgery, cardiac transplantation), urgent surgery, previous cardiac surgery, combined surgery (combination of more than one type of surgery), and complex surgery (*i.e.*, heart valve surgery with myocardial revascularization, double- or triple-valve surgery, or aortic root surgery). TRACK and TRUST were calculated after filling out forms and revising data using a Microsoft® Excel® spreadsheet.

Mann-Whitney U test compared TRACK and TRUST scores. Hosmer-Lemeshow goodness of fit test assessed calibration of these scores. This test compared the observed and expected transfusion using a logistic regression model, considering blood transfusion as a response and the score as independent variable. Accuracy was calculated using the area under the receiver operating characteristic curve (AUC) and was based on the sensitivity. The level of significance considered in all tests was 5%.

## RESULTS

Out of the 532 patients assessed, 34 were excluded due to inconsistent or incomplete data; therefore, the final sample was composed of 498 patients. Demographic and clinical profiles of patients are described in Table 1.

**Table 1:** Patients’ demographic and clinical profile (n = 498).

Variables	n (%) or mean±SD
Male sex	302 (60.6)
Age, years	56.3±14.6
Body area index, Kg/m2	28.5±12.4
Body surface area, m2	1.74±0.21
Diabetes mellitus	148 (29.7)
Hypertension	325 (65.3)
Preoperative Ht, %	33.9±6.5
Preoperative Hb (n = 497), g/100 ml	11.3±2.2
Preoperative creatinine, mg/dl	1.2±0.9

Hb=hemoglobin; Ht=hematocrit; SD=standard deviation

The distribution of types of surgery is presented in Table 2. Characteristics of proceedings and the calculated risk score are presented in Table 3.

**Table 2:** Types of cardiac surgery.

Type of surgery	n (%)
Myocardial revascularization	203 (41)
Valvular	188 (38)
Transplantation	42 (8)
Aortic root surgery	30 (6)
Combined surgery	24 (5)
Other	11 (2)

**Table 3:** Characteristics of surgeries analyzed, mortality rate, and risk scores calculated (TRUST and TRACK) for 498 patients.

Variables	n (%) or mean±SD
Previous cardiac surgery	36 (7.2)
Urgent surgery	18 (3.6)
CPB use	482 (96.8)
Period of CPB (n = 482), minutes	96.4±41.6
Anoxia (n = 470), minutes	67.4±47.8
Use of TRBCC	289 (58.0)
Blood bags/patient (n = 289)	
Up to one bag	106 (36.7)
Two bags	104 (35.9)
Three or more bags	79 (27.3)
Drained blood volume at postoperative period (n = 458), ml	610±416.6
Deaths	37 (7.4)
TRUST	2.3±1.1
TRUST categories	
Baseline	13 (2.6)
Low	109 (21.9)
Intermediate	171 (34.3)
High	134 (26.9)
Very high	71 (14.3)
TRACK	11.9±7.3

CPB=cardiopulmonary bypass; SD=standard deviation; TRACK=Transfusion Risk and Clinical Knowledge; TRBCC=transfusion of red blood cell concentrates; TRUST=Transfusion Risk Understanding Scoring Tool

Tables 4 and 5 demonstrate the observed and expected transfusion using TRUST and TRACK scores, respectively. According to these tables, only TRACK demonstrated a good calibration (*P*=0.238). Considering the TRUST score, the hypothesis was rejected (*P*=0.034).

**Table 4:** Observed and expected transfusion using TRUST score as predictor in the Hosmer-Lemeshow test.

	TRBCC = No		TRBCC = Yes		Patients
	Observed	Expected	Observed	Expected	
Baseline risk	12	8.519	1	4.481	13
Low risk	61	60.390	48	48.610	109
Intermediate risk	65	76.617	106	94.383	171
High risk	52	46.443	82	87.557	134
Very high risk	19	17.031	52	53.969	71

Chi-squared test = 8.64 (*P*=0.034).

TRBCC=transfusion of red blood cell concentrates; TRUST=Transfusion Risk Understanding Scoring Tool

**Table 5:** Observed and expected transfusion using TRACK score as predictor in groups defined in the Hosmer-Lemeshow test.

	TRBCC = No		TRBCC = Yes		Patients
	Observed	Expected	Observed	Expected	
1	41	41.902	23	22.098	64
2	27	22.842	11	15.158	38
3	21	25.430	26	21.570	47
4	27	25.980	26	27.020	53
5	24	22.604	27	28.396	51
6	16	18.103	30	27.897	46
7	16	21.139	46	40.861	62
8	20	15.257	34	38.743	54
9	14	10.762	35	38.238	49
10	3	4.981	31	29.019	34

Chi-squared test = 10.39 (*P*=0.238).

TRACK=Transfusion Risk and Clinical Knowledge; TRBCC=transfusion of red blood cell concentrates

**Fig. 1:**
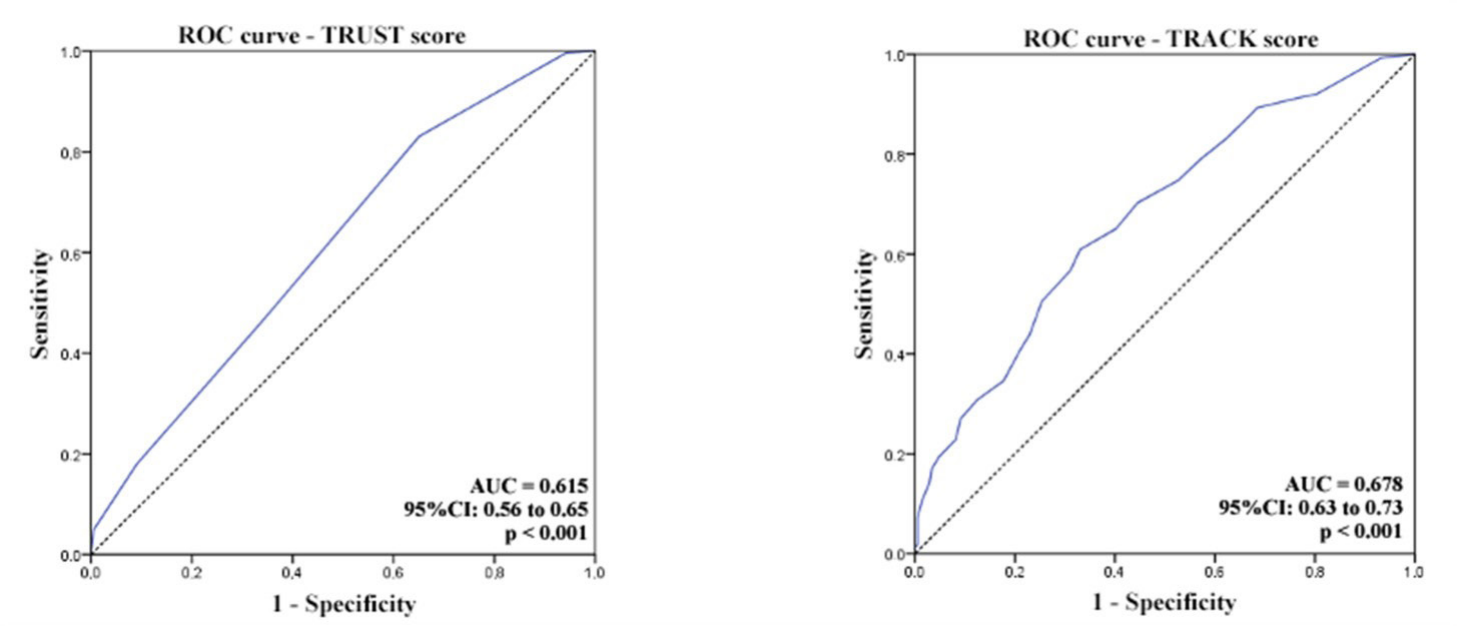
Receiver operating characteristic (ROC) curves and respective area under the ROC curve (AUC) of Transfusion Risk Understanding Scoring Tool (TRUST) and Transfusion Risk and Clinical Knowledge (TRACK) scores. CI=confidence interval.

The AUC for TRUST score was 0.615 (95% confidence interval [CI]: 0.56 to 0.65; *P*<0.001), whereas AUC for TRACK score was 0.678 (95% CI: 0.63 to 0.73; *P*<0.001). Although TRACK presented results slightly superior to TRUST, both scores presented a low accuracy (*i.e., P*<0.7) (Figure 1).

The best cutoff point found for TRUST was ≥ 1.5 (*i.e.*, values of ≥ 1.5 present a high risk to TRBCC) with sensitivity of 0.83 and specificity of 0.35. For the TRACK score, the cutoff point was ≥ 12 (sensitivity of 0.61 and specificity of 0.67).

**Fig. 2:**
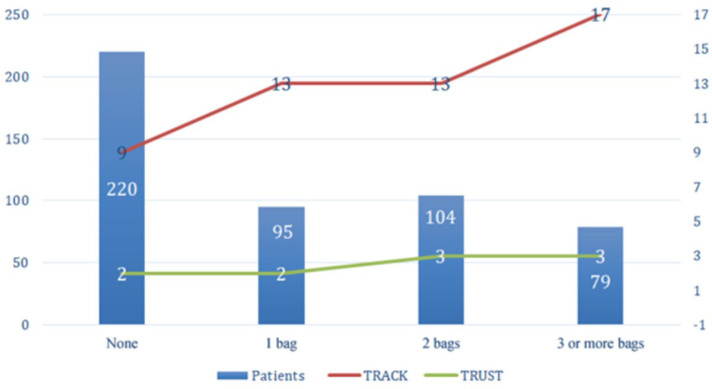
Transfusion Risk Understanding Scoring Tool (TRUST) and Transfusion Risk and Clinical Knowledge (TRACK) scores compared with number of blood bags. Kruskal-Wallis test, P-value < 0.001.

We also observed a significant association between high scores and the number of blood bags used, as shown in Figure 2 and Table 6.

**Table 6:** TRUST score categories vs. number of blood bags.

		Number of blood bags used					Total
			None	One	Two	Three or more	
TRUST risk	Baseline	N	12	0	0	1	13
		%	5.5%				
	Low	N	63	25	12	9	109
		%	28.6%	26.3%	11.5%	11.4%	21.9%
	Intermediate	N	69	42	37	23	171
		%	31.4%	44.2%	35.6%	29.1%	34.3%
	High	N	57	20	34	23	134
		%	25.9%	21.1%	32.7%	29.1%	26.9%
	Very high	N	19	8	21	23	71
		%	8.6%	8.4%	20.2%	29.1%	14.3%
Total		N	220	95	104	79	498
		%	100%	100%	100%	100%	100%

*P*-value = 0.001

## DISCUSSION

Risk scores are important management instruments in medicine. Many risk scores are used in cardiology, such as the Framingham, CHAD2DS2-VASc, and CRUSADE scores. The former stratifies the individual cardiovascular risk and suggests levels of investigation for cardiac and vascular diseases. The CHAD2DS2-VASc score calculates the risk of cardioembolism in patients with atrial fibrillation and suggests anticoagulation strategies, whereas the CRUSADE score predicts survival of patients with myocardial infarction without supra ST and impacts the guideline of care to patients with acute coronary syndrome^[^14^-^16^]^.

Predicting the risk of blood transfusion leads to clinical and economic implications. Previous studies in the United States of America demonstrated a financial impact of $4,000 to $10,000 dollars due to blood transfusions in cardiac surgeries^[^17^,^18^]^. Regarding clinical application, the use of hemoderivatives is associated with duration of mechanical ventilation, increased time of hospitalization and intensive care unit, and risk of infection^[^3^,^19^]^. In underfunded public health systems, such as the Brazilian public health system, this instrument identifies the population that most benefits from the allocation of resources.

The TRUST score was created in Toronto (Canada), whereas the TRACK score was developed in Italy and validated in England, United States of America, and India^[^10^,^16^,^17^]^. To our knowledge, no study validated instruments for the prediction of blood transfusion in the Brazilian population.

Logistic regression is the standard statistical analysis to assess the effects of multiple risk factors in a binary variable, such as blood transfusion risk scores. The accuracy of the model is determined using discrimination and calibration. Calibration measures the ability of the score to predict the observed result. The most used method is the Hosmer-Lemeshow goodness of fit test. The statistical significance implicates that the model is not calibrated^[^14^]^. In this study, although TRACK and TRUST consider similar characteristics of patients, only the former demonstrated good calibration (*P*=0.238 *vs.* TRACK *P*=0.034) for predicting TRBCC after cardiac surgery.

For TRUST calculation, one point is attributed for each factor: Hb < 13.5 mg/dl, weight < 77 kg, female sex, age > 65 years, non-elective surgery, creatinine > 1.36 mg/dl, previous cardiac surgery, and combined surgery^[^6^]^. In contrast, TRACK considers six points for age, two points for weight < 60 kg (female) and < 85 kg (male), four points for female sex, seven points for complex surgery, and one point for each percentage point of Ht < 40%^[^5^]^. The different weights considered for Ht (or Hb) could justify differences between scores in the population studied.

The discrimination of the test measures how well a model distinguishes patients from needing or not hemoderivatives in the postoperative period of cardiac surgery. This discrimination is measured using the AUC. TRUST and TRACK demonstrated significant accuracy and could discriminate the need for blood transfusion (AUC > 0.5). However, this ability was considered low (AUC < 0.7)^[^15^]^. We found an AUC of 0.678 (0.630 to 0.730) for TRACK, close to values of the Italian (0.710 [0.681 to 0.724]) and British (0.710 [0.710 to 0.720]) studies. AUC was 0.768 (0.750 to 0.785) in the American study, whereas the Indian study reported 0.756 (0.729 to 0.782)^[^5^,^10^,^16^,^17^]^. This comparison showed that the power of discrimination in the Brazilian population was worse than in other countries.

Some factors may justify these results, such as differences between blood transfusion protocols^[^13^]^ and nutritional status of the population. In the study conducted in Toronto, patients presented a mean Hb of 13.4 (± 1.55) mg/dl, whereas we found a value of 11.3 (± 2.2) mg/dl^[^6^]^. In another study, patients submitted to cardiac surgery using cardiopulmonary bypass in Portugal demonstrated a mean preoperative Ht of 41% (± 4.4), whereas our sample demonstrated 33.9% (± 6.5)^[^10^]^.

This factor may also explain the fact that 58% of patients received at least one bag of red blood cell concentrates. This number is higher than in other studies. In England, a study conducted with more than 19,000 patients evaluated preoperative anemia in cardiac surgery and demonstrated a blood transfusion rate of 45.1%. Among anemic patients (males with Hb < 13 mg/dl and females with Hb < 12 mg/dl), blood transfusion rate was 63.9%^[^19^]^. In a study conducted with more than 10,000 patients at the Cleveland Clinic (United States of America), the prevalence of anemia was 26%; among these, 66.59% required blood transfusion^[^20^]^. Another American cohort study considering 798 different hospitals with more than 100,000 patients submitted to myocardial revascularization presented a blood transfusion rate of 56.1%. Nevertheless, this rate varied widely between hospitals (7.8% to 92.8%)^[^21^]^. In the Indian study performed with more than 1,000 patients, blood transfusion rate was 76.2%^[^17^]^. This worldwide variability in blood transfusion was already demonstrated in an international multicentric study involving 5,436 patients from 16 countries in North America, South America, Europe, Middle East, and Asia: perioperative and postoperative blood transfusion varied between 9% and 100% and between 25% to 87%, respectively^[^22^-^24^]^.

The mean Hb (11.3 mg/dl) and Ht (33.9%) of patients from our database suggest that patients were operated with anemia, according to the World Health Organization^[^25^]^. This characteristic differed from a cohort conducted in São Paulo with 1,490 patients (mean Ht of 39.39%)^[^29^]^. These data corroborate with findings of a Brazilian study with more than 8,000 adult patients, evidencing the high prevalence of anemia in Brazilian residents of north and northeast regions^[^30^]^.

The blood loss found in our study was similar to that observed in a reference center in Brazil (610±416.6 ml *vs.* 610±600 ml) and Germany (549±941 ml)^[^25^,^26^]^. Although heavy bleeding and reoperation due to bleeding impact on cardiac surgery, we believe that blood loss did not influence the low accuracy^[^33^]^.

High scores were also associated with increased use of hemoderivatives. The absence of this relationship was criticized in other validation studies and studies that created other risk scores for blood transfusion^[^7^,^14^]^. Despite associations, the cutoff point found was very different from other validation studies. For example, the best value found in the American study that validated TRACK was 22 (*i.e.*, TRACK scores > 22 presented 92% risk of receiving a blood transfusion), whereas we found a cutoff point of 12 with sensitivity of 0.61 and specificity of 0.67^[^31^-^34^]^.

### Limitations

Our study has some limitations. First, the use of other hemoderivatives was not analyzed, such as platelets or fresh plasma. Furthermore, the study was conducted in a single center and could not necessarily reflect the national reality. Although we used a small sample size compared with other international validation studies, the Hosmer-Lemeshow goodness of fit test has limited validity in large samples. Moreover, considering that power of this test increases with sample size, small discrepancies between estimates of a model and actual probabilities in a large dataset would probably lead to rejection of the null hypothesis, even if such discrepancies were irrelevant to the test^[^35^]^. We suggest future multicentric validation studies or creating a specific score considering the typical characteristics of the Brazilian population.

## CONCLUSION

Between the scores analyzed, only the TRACK score showed a good calibration, but low accuracy, to predict postoperative TRBCC after cardiac surgery in patients from northeastern Brazil.
